# Acetaldehyde Content and Oxidative Stress in the Deleterious Effects of Alcohol Drinking on Rat Uterine Horn

**DOI:** 10.1155/2013/161496

**Published:** 2013-11-17

**Authors:** Lara Romina Buthet, María Eugenia Maciel, Leandro Néstor Quintans, Carmen Rodríguez de Castro, Martín Hernán Costantini, Silvia Laura Fanelli, José Alberto Castro, Gerardo Daniel Castro

**Affiliations:** ^1^Centro de Investigaciones Toxicológicas (CEITOX-UNIDEF, MINDEF-CONICET), Juan B de La Salle 4397, B1603ALO Villa Martelli, Argentina; ^2^Instituto de Investigación e Ingeniería Ambiental, Universidad Nacional de General San Martín, Avenue 25 de Mayo y Francia, 1650 San Martín, Argentina

## Abstract

After alcohol exposure through a standard Lieber and De Carli diet for 28 days, a severe atrophy in the rat uteirne horn was observed, accompanied by significant alterations in its epithelial cells. Microsomal pathway of acetaldehyde production was slightly increased. Hydroxyl radicals were detected in the cytosolic fraction, and this was attributed to participation of xanthine oxidoreductase. They were also observed in the microsomal fraction in the presence of NADPH generating system. No generation of 1-hydroxyethyl was evidenced. The *t*-butylhydroperoxide-induced chemiluminescence analysis of uterine horn homogenates revealed a significant increase in the chemiluminiscence emission due to ethanol exposure. In the animals repeatedly exposed to alcohol, sulfhydryl content from uterine horn proteins was decreased, but no significant changes were observed in the protein carbonyl content from the same samples. Minor but significant decreasing changes were observed in the GSH content accompanied by a tendency to decrease in the GSH/GSSG ratio. A highly significant finding was the diminished activity content of glutathione peroxidase. Results suggest that acetaldehyde accumulation plus the oxidative stress may play an additional effect to the alcohol-promoted hormonal changes in the uterus reported by others after chronic exposure to alcohol.

## 1. Introduction

In previous studies from our laboratory we reported that the rat uterine horn has different metabolic pathways able to generate acetaldehyde from ethanol accompanied by a low capacity to destroy it through aldehyde dehydrogenase (ALDH). That led to long lasting increases in acetaldehyde levels in the uterus during ethanol exposure [1].

The identified enzymes involved in acetaldehyde production included alcohol dehydrogenase (ADH) and xanthine oxidoreductase (XO) in the cytosolic fraction and the flavoenzyme NADPH oxidase and catalase in the microsomal fraction [1]. The ALDH activity present in the mitochondrial fraction was almost negligible and not detectable at all in the microsomal or cytosolic fraction. That was envisaged to be a reasonable explanation for the observed acetaldehyde increased levels observed [1]. However, other enzymatic systems and cofactors such as GSH and glutathione transferase (GST) that are also able to handle acetaldehyde to eliminate it were not determined in those studies.

Notwithstanding, acetaldehyde is not the only harmful metabolite produced during interaction of ethanol with enzymes and cellular fractions in different tissues. In effect, previous studies from our and other laboratories in tissues such as liver, mammary tissue, prostate, and testes evidenced the formation of free radicals of different structure. The observed reactive deleterious reaction products included hydroxyl, 1-hydroxyethyl, and acetyl radicals [2–8]. Free radicals are able to attack DNA, lipids and proteins, and other metabolically relevant molecules and cause cell injury, promote cancer, or cause different pathologies [9].

In the present work we attempted to enhance our knowledge about the harmful effects of alcohol drinking on the rat uterine horn, directed to shed some additional light on the reasons for the severe morphological changes observed during our studies.

These studies include, besides morphological and ultrastructural observations, attempts to detect the formation of free radicals and whether oxidative stress was promoted during repetitive alcohol drinking. 

## 2. Materials and Methods

### 2.1. Chemicals

Ethanol (analytical grade) was from Sintorgan (Villa Martelli, Argentina). Acetaldehyde was from Fluka (Buchs, Switzerland). Hypoxanthine, allopurinol, NAD^+^, and NADP^+^ were from Sigma Chemical Co. (St. Louis, USA). All other chemicals were of the best quality available. Liquid diet ingredients were Lieber-De Carli Regular, no. 710260, for rodents ethanol group and Lieber-De Carli Regular, no. 710027, for rodents control group; both were purchased from Dyets, Inc. (Bethlehem, PA, USA).

### 2.2. Animals and Treatments

Noninbred Sprague Dawley rats were used in our studies. The procedures employed for breeding, housing, and handling animals were established by the Food, Drug, and Medical Technology National Administration (ANMAT, Buenos Aires, Argentina). The starting breeding colony was from Charles River (Wilmington, USA). For the studies on the metabolism to free radicals in microsomal and cytosolic fractions, Sprague Dawley female rats (220–250 g body weight) were used. Food was withdrawn 12–24 h before sacrifice, but the animals had free access to water. 

In the treatment with the ethanol containing liquid diet, Sprague Dawley female rats (125–150 g body weight, 5-6 weeks age) were fed *ad libitum* for 28 days with a nutritionally adequate liquid diet (Lieber and De Carli Regular rodent diet) [10, 11]. The liquid diet used provided 1 kcal mL^−1^, where 35% calories derived from fat, 47% from carbohydrate (maltose-dextrin), and 18% from protein. The rats were housed in individual cages and separated into two dietary groups: ethanol group (EtOH treated) and control group (Control). Both groups were pair fed with the same diet except that in the alcoholic, ethanol provided 36% of the calories, replacing isocaloric amount of carbohydrate. These diets assured continued growth in all animals and normal liver in the control, whereas in the rats fed with alcohol, fatty liver has developed. A record of daily liquid diet consumption, using a graduated feeding tube (Dyets, Inc. Catalog no. 900006), was made, and their body weight changes were registered. It was started with 30 gL^−1^ ethanol of the liquid diet for two days and 40 gL^−1^ for the subsequent two days followed by the final formula containing 50 gL^−1^ during 24 additional days. The amount consumption was 13–15 g ethanol kg^−1^ per day. The animals were sacrificed by decapitation with a Harvard guillotine and bled, to minimize the potential interference of hemoglobin. Uterine horn and liver tissues were rapidly excised and processed.

### 2.3. Isolation of Uterine Horn Tissue Cytosolic and Microsomal Fractions

Animals were killed by decapitation, and their uterine horns were rapidly excised, separated from ovary and oviduct, and processed to obtain cytosolic and microsomal fractions. Cytosolic and microsomal fractions were obtained from whole uterine horn tissue homogenates by cellular fractionation procedures via ultracentrifugation at 4°C. Liver microsomes and cytosol were obtained by the same procedure [12].

### 2.4. Ethanol Metabolism to Acetaldehyde in the Microsomal Fraction

Preparations containing microsomes (0.17–0.19 mg protein/mL), NADPH generating system (0.45 mM NADP^+^, 4 mM d,l-isocitric acid trisodium salt, and 0.25 units of isocitric dehydrogenase), and 0.14 M ethanol in 50 mM KH_2_PO_4_, pH 7.4 (3 mL final volume) were incubated for 1 h at 37°C under air. Three samples per group were run, each consisting of microsomes from a separate lot of pooled uterine horn tissue (four animals each). Incubations were performed in aluminum-sealed neoprene-septum-stoppered glass vials. The reaction was terminated by plunging in ice. After adding 1 mL of saturated NaCl solution, samples were kept at 37°C for 15 min, and an aliquot (100 **µ**L) of the headspace was analyzed by GC-FID. Chromatographic conditions were as follows: column, GS-Q PLOT, 25 m × 0.53 mm i.d. (J & W Scientific, CA); temperature, 11°C isothermal; injection port temperature, 150°C, FID: 200°C [1].

### 2.5. Ethanol Metabolism to Acetaldehyde in the Cytosolic Fraction

Incubation mixtures containing cytosol (1.4–1.6 mg protein/mL) in STKM buffer (0.25 M sucrose/50 mM Tris-HCl, pH 7.5/2.5 mM KCl/5 mM MgCl_2_), 0.25 mM hypoxanthine, 0.3 mM NAD^+^, and 0.14 M ethanol (3 mL final volume) were conducted for 1 hour at 37°C under air atmosphere. Three samples per group were run, each consisting of cytosol prepared from a separate lot of pooled uterus tissue (four animals each). Incubations were performed in aluminum-sealed neoprene-septum-stoppered glass vials (15 mL). Samples were processed as described above. Acetaldehyde was quantified in the head space by GC-FID in the same conditions as above [1].

### 2.6. Hydroxyl and 1-Hydroxyethyl Radicals Determination in “*In Vitro*” Biological Systems

The incubation mixture contained microsomes (uterine horn: 2.4 ± 0.4 mg prot/mL; liver: 4.1 ± 0.5 mg prot/mL), 18.8 mM PBN, 0.2 M ethanol, and NADPH generating system in a buffered media (50 mM phosphate buffer, pH 7.4). Control samples without ethanol or NADPH generating system were run simultaneously. In the case of cytosol (uterine horn: 6.0 ± 0.3 mg prot/mL; liver: 5.3 ± 0.5 mg prot/mL), incubation mixture contained 9.4 mM PBN, 0.25 mM hypoxanthine, and 0.15 mM allopurinol. Control samples were run accordingly.

After incubating for 1 hour at 37°C, the reaction volume (3 mL) was extracted with 500 **µ**L toluene and centrifuged, and the organic layer was evaporated under nitrogen. The residue was silylated with BSTFA : acetonitrile (1 : 1), 60°C, 15 min and analyzed by GC-MS-SIM. Selected ion monitoring (SIM) of mass spectrum of adducts was employed to increase sensitivity. Selected masses were 250 (M—^•^CHCH_3_OTMS) and 194 (*m/z* 250—C_4_H_8_). Dwell time was 50 ms for both masses selected.

Chromatographic conditions were as follows: column, 5% phenylmethyl silicone, 12 m × 0.2 mm i.d., programmed from 100°C to 300°C at a ramp of 10°C/min. Injection port was at 250°C and transfer line to MS, 300°C [8].

### 2.7. Determination of *t*-Butylhydroperoxide-Induced Chemiluminescence in Rat Uterine Horn Tissue Homogenates

Chemiluminiscence was measured in a Wallac-Rack Beta liquid scintillation counter at room temperature in an out of coincidence mode [12–14]. Rat uterine horn tissue was homogenized (*≈*7 mg protein/mL) in 0.25 M sucrose, 50 **µ**M deferoxamine in TKM buffer (50 mM Tris-HCl, 5 mM MgCl_2_, and 2.5 mM KCl), and pH 7.5 in flasks that were kept at 37°C for 10 min in a Dubnoff shaker. Chemiluminiscence measurement was started by addition of 3 mM *t*-butylhydroperoxide (TBHP). Three samples per group were run, each consisting of a homogenate from a separate lot of pooled uterine horn tissue (five animals each).

### 2.8. Protein Carbonyl Content Determination

Rat uterine horn tissue was homogenized in 0.15 M Tris-HCl, pH 7.4, and 1 mM KH_2_PO_4_. Protein carbonyl was carried out in 600 ×g supernatants by the 2,4-dinitrophenylhydrazine technique [15]. Three samples per group were run, each consisting of a homogenate from a separate lot of pooled uterine horn tissue (five animals each). Carbonyl content was calculated from the absorbance at 370 nm, using a molar absorption coefficient of 22,000 M^−1^ cm^−1^ [16].

### 2.9. Protein Sulfhydryl Content Determination

Rat uterine horn tissue was homogenized as described in protein sulfhydryl determination. Protein sulfhydryl determination was carried out in 600 ×g supernatants using Ellman's reagent according to the Jocelyn technique [15]. Three samples per group were run, each consisting of a homogenate from a separate lot of pooled uterine horn tissue (five animals each). Sulfhydryl content was calculated from the absorbance at 412 nm, using a molar absorption coefficient of 13,100 M^−1^ cm^−1^ [17].

### 2.10. Determination of Total and Reduced Glutathione Content

Reduced and oxidized glutathione levels were measured by the method of Venturino et al. [18]. Tissue samples were frozen in liquid nitrogen and then pulverized in a steel mortar. Then they were homogenized in 2.5 parts of water and 2.5 parts of 10% TCA (in that order) and centrifuged at 10,000 ×g for 10 min. at 4°C. The volume of supernatant obtained was measured, recorded, and placed in 450 **µ**L aliquots into two conical tubes (for total glutathione and reduced glutathione analysis). Then, each tube was added of 400 **µ**L of neutralization buffer (500 mM phosphate, pH 8). In the tube for total glutathione measurement, 25 **µ**L of NADPH (4.2 mM in reaction buffer) and 25 **µ**L of glutathione reductase (24 U/mL in reaction buffer) were added. In the tube for reduced glutathione, the volume was completed with reaction buffer. After stirring and incubating for 30 min at 25°C, both conical tubes were added of 900 **µ**L of 10% TCA, then both were stirred and centrifuged at 10,000 ×g for 10 min. Samples (40 **µ**L of supernatant) were added to 180 **µ**L of DTNB (1.67 mM in sample buffer) and read by triplicate at 414 nm in a Beckman Coulter microplate reader.

### 2.11. Assay of Glutathione S-Transferase Activity (GST)

GST activity was assayed by the method of Habig et al. [19]. The reaction mixture (200 **µ**L) consisted of 0.1 M potassium phosphate (pH 6.5), 0.1% triton X-100, 1 mM GSH, and tissue cytosol. Assays were conducted at 25°C (room temperature). The reaction was initiated by addition of 1 mM 1-chloro-2,4-dinitrobenzene (CDNB). A complete assay mixture without enzyme was used as control. The thioether formation was determined by reading the absorbance at 340 nm, and quantification was performed using the molar extinction coefficient of CDNB (9.6 mM^−1^ cm^−1^). The enzymatic activity was expressed as nmol of glutathione conjugated formed per min per mg of protein.

### 2.12. Assay of Glutathione Reductase Activity (GRed)

GRed activity was measured by a modification of the method of Carlberg and Mannervik [20]. The reaction mixture (200 **µ**L) consisted of 0.1 M potassium phosphate (pH 7.6), 0.1 mM NADPH, 0.5 mM EDTA, 1 mM GSSG, and tissue cytosol. The enzyme activity was determined by measuring the disappearance of NADPH at 340 nm and was expressed as nmol NADPH oxidized per min per mg protein.

### 2.13. Assay of Glutathione Peroxidase Activity (GPx)

GPx activity was assayed by a modified version of the method of Flohé and Günzler [21], adapted for use in a microplate reader. Tissue cytosol was added to the reaction mixture and incubated at 37°C for 10 min. The reaction was initiated by the addition of 20 **µ**L of 1.5 mM H_2_O_2_. The absorbance change was followed at 340 nm for 3 minutes. The nonenzymatic reaction rate is correspondingly assessed by replacing the cytosol by buffer. The molar extinction coefficient of NADPH (6.22 mM^−1^ cm^−1^) was used to determine the activity of GPx.

### 2.14. Transmission Electron Microscopy

Ten female rats per group (Control and EtOH treated animals) were anesthetized by diethyl ether. The estrus cycle was followed in each animal by observing the changes in types of cells from vaginal smears. The uterine horn was rapidly removed and immediately placed in chilled 2% formaldehyde : 2% glutaraldehyde in 100 mM cacodylate buffer containing 0.02% CaCl_2_, pH 7.4 and promptly cut under the fixative. After adequate fixation, 10 cubes (1 mm^3^) per each rat uterine horn were washed with barbital buffer and post fixed with 1% osmium tetroxide. Then, they were stained as a whole with uranyl acetate, dehydrated with graded ethanol, and embedded in epoxy resin. Sections 1 **µ**m thick were stained with toluidine blue and examined with a light microscope in order to select epithelial areas for thin sectioning. Thin sections were cut with a diamond knife and mounted on copper grids (300 mesh), stained with uranyl acetate and lead citrate, and examined in a Philips EM300 transmission electron microscope [22, 23]. The microscopic observation of the uterine horn sections was carried out in a blinded fashion by two observers who were unaware of the treatment group. The photomicrographs were representative average of observations.

### 2.15. Protein Concentrations

Protein concentrations were determined by the method of Lowry et al., using bovine serum albumin as standard [24].

### 2.16. Statistics

Statistical analysis was carried out by ANOVA, unpaired *t*-test (Student's *t*-test), or the Welch test. Calculations were performed using GraphPad Software and Microsoft Office Excel. Differences were considered significant when *P* < 0.05 [25].

## 3. Results

### 3.1. Morphological Changes in the Uterine Horns from Rats that Received the Alcohol Containing Liquid Diet during 28 Days

**Figure 1:**
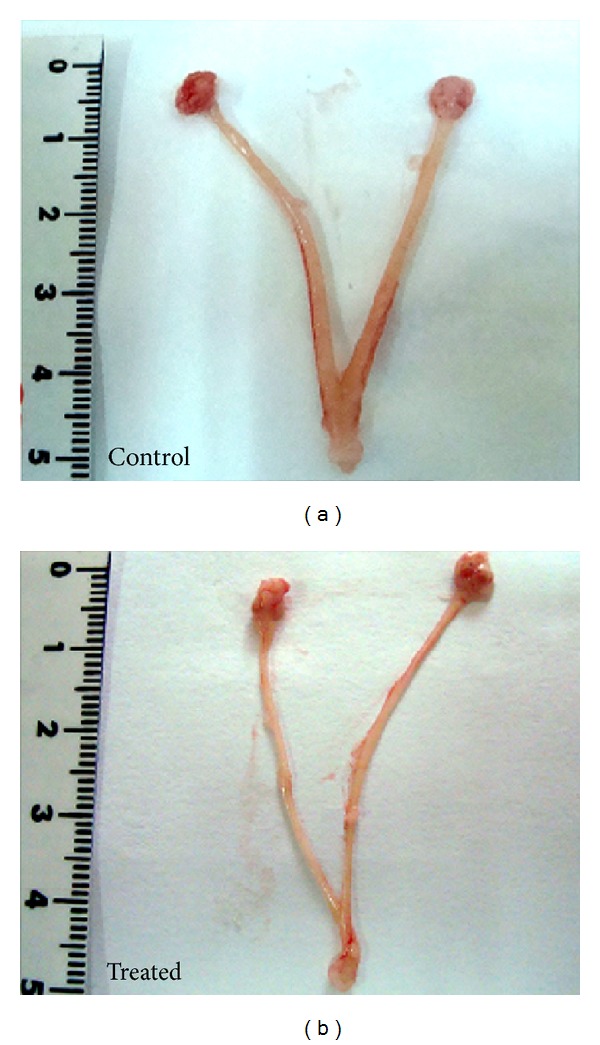
Representative samples from animals being at the proestrous stage of the estral cycle. Morphological observations in reproductive organs from rats receiving an alcohol containing liquid diet during 28 days.

Representative samples of reproductive organs from ethanol-treated and control animals are depicted in Figure 1. Both were representative examples from animals being at the proestrous stage of the estral cycle.

Body weight gain of both groups at the end of the experiment was not significantly different, but, in contrast, there were major differences in the uterine horn weights themselves between the alcohol-treated group and the control one (Table 1).

### 3.2. Generation of Hydroxyl or 1-Hydroxyethyl Free Radical Species during the Uterine Horn or Liver Alcohol Metabolism in Their Microsomal and Cytosolic Fractions

**Figure 2:**
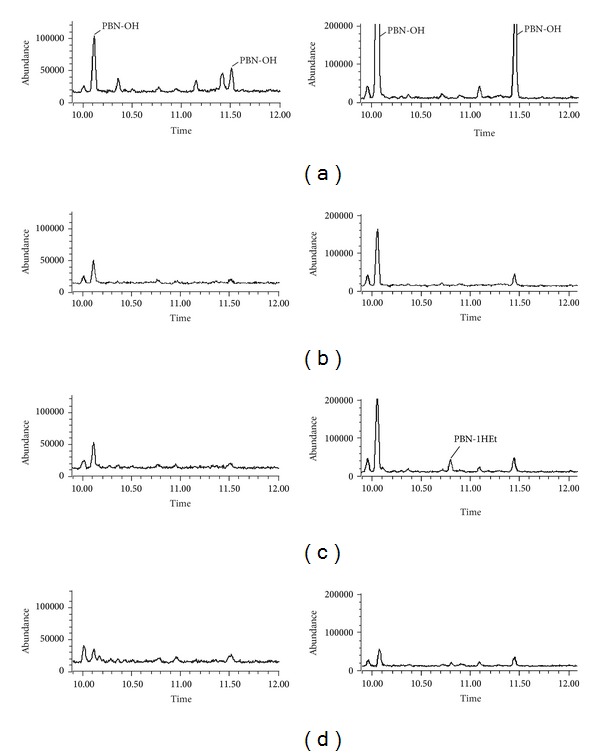
Selected-ion current profile obtained from GC-MS-SIM analysis of a sample from incubation containing microsomal fraction and ethanol, in the presence of the spin trap PBN, after trimethylsilylation. Left: uterine horn microsomes. Right: liver microsomes. (a) Microsomes + NADPH generating system. (b) Microsomes only. (c) Microsomes + NADPH generating system + ethanol. (d) Microsomes + ethanol.

Via PBN spin trapping of radicals coupled to GC-MS analysis the generation of hydroxyl radicals was detected during the NADPH and oxygen-dependent uterine horn microsomal metabolism of ethanol. In contrast, both hydroxyl and 1-hydroxyethyl radicals were detected when liver microsomes were used instead (see Figure 2).

**Figure 3:**
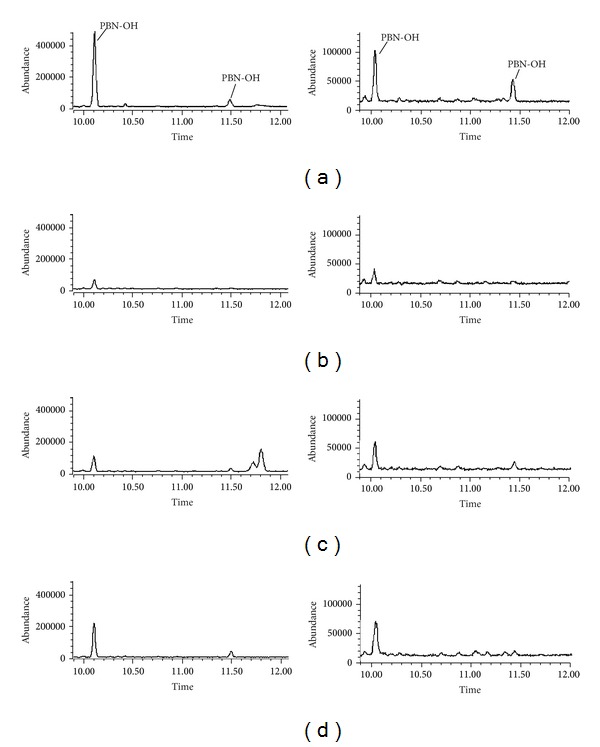
Selected-ion current profile obtained from GC-MS-SIM analysis of a sample from incubation containing cytosolic fraction and ethanol, in the presence of the spin trap PBN, after trimethylsilylation. Left: uterine horn cytosol. Right: liver cytosol. (a) Cytosol + hypoxanthine. (b) Cytosol + hypoxanthine + allopurinol. (c) Cytosol + hypoxanthine + ethanol. (d) Cytosol only.

The cytosolic fractions from both, the uterine horn and liver, were able to generate hydroxyl radical species in the presence of hypoxanthine as cofactor. The generation process was less intense in liver than in the uterine horn derived samples, and in both cases the processes was fully inhibited by allopurinol. No generation of 1-hydroxyethyl radicals was observed (see Figure 3).

### 3.3. Ethanol Metabolism to Acetaldehyde in the Uterine Horn Microsomal Fraction from Rats Receiving an Alcohol Containing Liquid Diet

Both a NADPH-dependent and a NADPH-independent microsomal metabolism of ethanol to acetaldehyde were observed (Table 2). The former was significantly more intense than the latter. After chronic ethanol drinking either the activity of the NADPH-dependent pathway or that in the absence of the cofactor was slightly but significantly enhanced.

### 3.4. Ethanol Metabolism to Acetaldehyde in the Uterine Horn Cytosolic Fraction from Rats Receiving an Alcohol Containing Liquid Diet

Repetitive ethanol drinking significantly changed the response of the cytosolic oxidation pathway from ethanol to acetaldehyde. In Control group, that activity was significantly enhanced by the presence of NAD^+^ or hypoxanthine + NAD^+^ in the incubation mixture. Allopurinol was able to inhibit the metabolism in all cases in this group. By contrast, in the EtOH-treated group, the presence of the cofactors NAD^+^ or hypoxanthine led to a strong depletion of acetaldehyde formation (see Table 3). 

### 3.5. *t*-Butylhydroperoxide-Induced Chemiluminiscence in Homogenates of Uterine Horn Tissue from Rats Receiving an Alcohol Containing Liquid Diet

**Figure 4:**
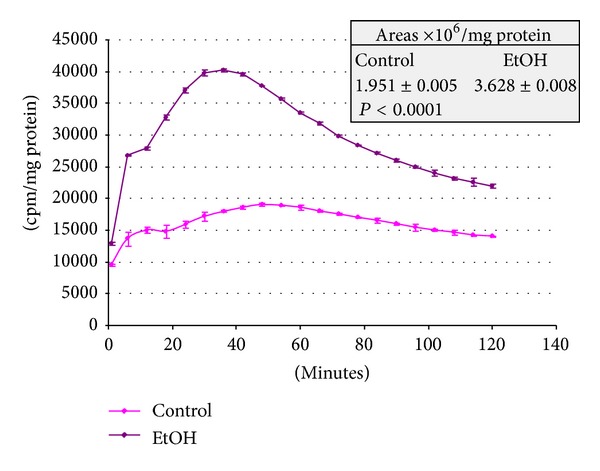
*t*-Butylhydroperoxide induced chemiluminiscence in rat uterine horn homogenates from rats receiving an alcohol containing liquid diet. Rat uterine horn tissue homogenized in 0.25 M sucrose, 50 M deferoxamine in TKM buffer, pH 7.5 (*≈*7 mg protein/mL) was kept at 37°C for 10 min in a Dubnoff shaker. Chemiluminiscence measurement was started by addition of 3 mM *t*-butylhydroperoxide. Values are the mean ± S.D. Three samples per group were run, each consisting of a homogenate from a separate lot of pooled uterine horn tissue (five animals each).

In our studies on the total hydroperoxide-induced chemiluminiscence emitted by uterine horn tissue homogenates, a significant difference in area and shape of curves was observed between Control and that from rats receiving the ethanol containing diet (Control: 1.951 ± 0.005 × 10^6^; EtOH: 3.628 ± 0.008 × 10^6^; *P* < 0.0001) (Figure 4).

### 3.6. Protein Sulfhydryl and Protein Carbonyl Content in Uterine Horn from Rats Receiving an Alcohol Containing Liquid Diet

The protein carbonyl content of uterine horn from animals receiving the alcohol containing liquid diet for 28 days was not significantly increased when compared to values obtained from samples derived from the control group. In contrast, the protein sulfhydryl content from uterine horn samples of the same animals revealed a significant decrease promoted by the alcohol drinking treatment (Table 4).

### 3.7. Effect of Repetitive Alcohol Drinking on Glutathione Levels in Uterine Horn and Liver Tissues

Repetitive alcohol drinking during 28 days led to different effects on glutathione levels in uterine horn tissue when compared to those from liver. While liver responded adaptatively to alcohol drinking by increasing their GSH levels, in contrast, uterine horn tissue GSH levels displayed minor but significantly decreasing changes (Table 5).

### 3.8. Glutathione S-Transferase Activity (GST), Glutathione Reductase Activity (GRed), and Glutathione Peroxidase Activity (GPx) in Uterine Horn and Liver Tissues from Rats Receiving an Ethanol Containing Liquid Diet

Both uterine horn and liver tissues exhibited a different response in their GST, GRed, and GPx activities after alcohol drinking during 28 days.

In liver, GST and GPx were not significantly modified by the repetitive alcohol drinking exposure. Notwithstanding, significant increases resulted in GRed activity under the same circumstances (Table 6).

In contrast, in the uterine horn tissue GRed did not change significantly, but, more important, highly significant increase was observed in the GST activity and a significant decrease was found in the GPx value (Table 6).

### 3.9. Ultrastructural Alterations in the Uterine Horn Tissue of Rats Treated with Ethanol Liquid Diet

Our structural studies in the uterine horn tissue from animals repetitively exposed to ethanol showed that alcohol drinking may lead to alterations in their epithelial cells. The ultrastructure of the uterine epithelial cells from control rats, observed in our studies, was similar to the one previously described by others [26, 27]. 

**Figure 5:**
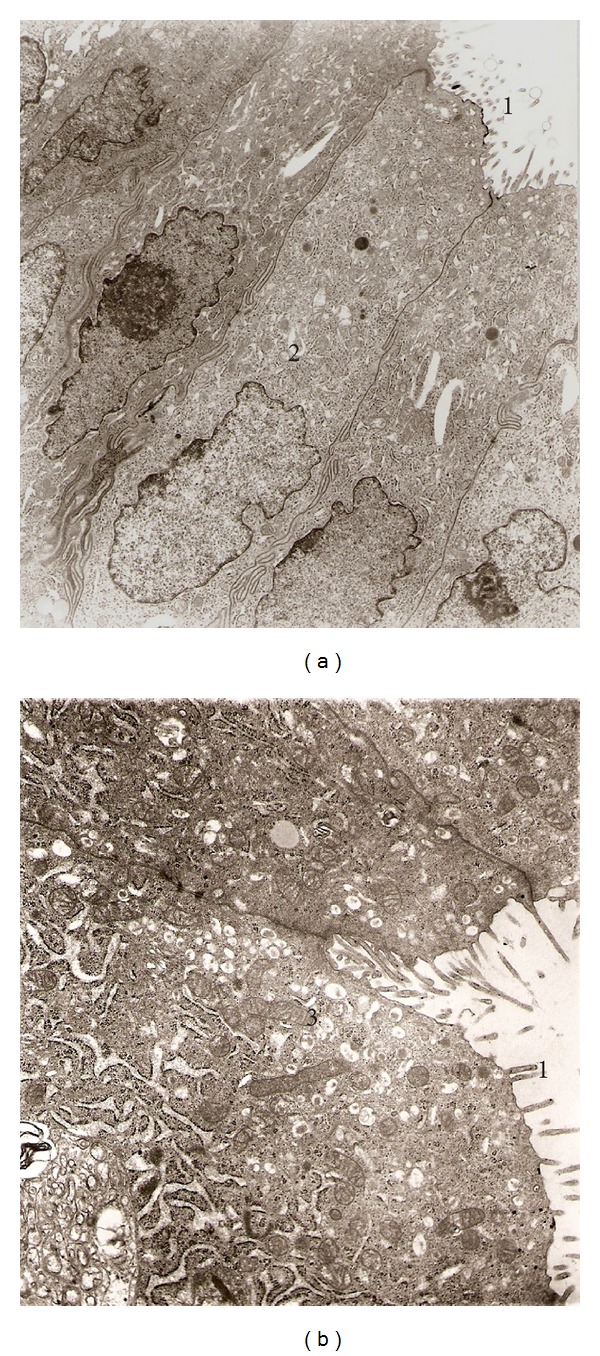
Electron micrographs from rat uterine horn (Control). (a) Epithelial cells showing microvilli facing to lumen (1) and normal nuclear membrane (2), 4,980x. (b) In addition numerous secretory granules and mitochondria can be observed also (3), 8,800x.

For example, in control rats, during early estrus, the surface epithelium is composed of a single layer of columnar secretory cells. The luminal cell surface is provided with strong microvilli. The nuclei of columnar cells, elongated, round, or irregular in shape, are uniformly situated. The cytoplasm has an abundance of ribosomes, some profiles of granular endoplasmic reticulum, and a few mitochondria. The uterine glands are simple, slightly branched tubular glands (Figure 5).

**Figure 6:**
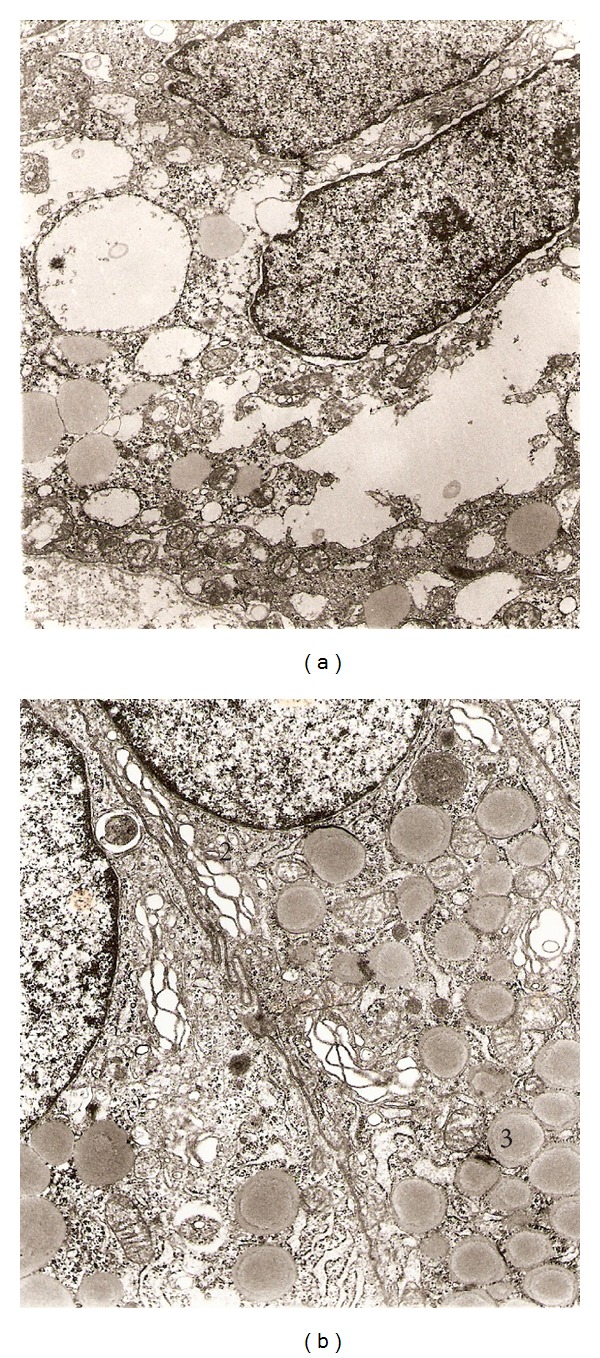
Electron micrographs from rat uterine horn (EtOH treated). (a) Epithelial cells show extended vacuolization, with nuclei having irregular shape and dilated perinuclear membrane (1), 8,800x. (b) Dilated Golgi complex (2) and numerous lipid drops in cytoplasm (3), 8,800x.

The repetitive administration of the Lieber and De Carli diet for 28 days produced severe ultrastructural alterations in the epithelial cells of the uterus horn. These alterations described here occurred irrespectively of the cycle stage of the animals at the time of their sacrifice. The endometrium evidenced dilated uterine glands with relatively scanty stroma. A prominent and dilated Golgi complex can be observed. Some areas showed vacuolar degeneration and increased glandular secretion with electron-lucent secretory droplets appearing in the apical region (see Figure 6).

## 4. Discussion

Epidemiological studies made in different countries evidenced that alcohol misuse among women is an important and growing problem. Further, the harmful consequences of alcohol misuse are particularly severe for women [28–33].

Our laboratory has focused its interest on the study of the deleterious consequences of women excessive alcohol drinking on the reproductive system and carcinogenesis. Ovarian and mammary tissues were already studied in more detail, and initial efforts directed to uterine horn were also performed [1, 23, 34, 35].

The present studies and results attempt to provide further information on the effects of alcohol drinking in the rat uterine horn.

The obtained results justified our interest. In effect, the mere macroscopic observation of the uterine horns from rats exposed to repetitive alcohol drinking revealed a very important decrease in the diameter of the uterine horns, from animals being at the same stage of the cell cycle (Figure 1). That effect can be quantified by the highly significant weight decrease observed in the uterine horns from alcohol drinking animals (Table 1).

The harmful effects of repetitive alcohol exposure on the uterine horns were accompanied by severe alterations in the ultrastructure of their cellular components when compared to that in control animals, as revealed by our electron microscopy studies depicted in Figures 5 and 6. In effect, columnar epithelial cells from the uterine horn mucosa exhibited marked alterations of their organelles. That included marked vacuolization and dilatation of the nuclei, endoplasmic reticulum, and Golgi apparatus membranes. There is a general disorganization of the cellular structure.

The wide derangement of the cellular structure observed in the rat uterine horn suggests the presence of a chemically induced cell injury process beyond the unavoidable hormone-mediated effects promoted by alcohol drinking. Not all the effects of alcohol drinking in different target organs can be explained in terms of endocrine disturbances [12, 23, 36].

In the uterine horn microsomal fraction from Control animals and from those receiving ethanol via the respective Lieber and De Carli diet for 28 days, the presence of a NADPH-dependent pathway of oxidation of ethanol to acetaldehyde was observed. A little but significant increase in ethanol metabolism was found by repetitive alcohol exposure (Table 2).

In the course of our previous studies we reported the presence in the uterine horn of cytosolic fraction metabolic pathways of transformation of alcohol to acetaldehyde mediated by xanthine oxidoreductase and also activity of ADH [1]. The participation of xanthine oxidoreductase to perform this metabolism should not be unexpected in light of the known lack of specificity exhibited by this enzyme in its electron transferring activity occurring at its molybdenum center [37]. The depletion exerted by NAD^+^ or hypoxanthine on the cytosolic metabolism of alcohol to acetaldehyde at the uterine horn suggests that repetitive alcohol exposure induces an enzyme able to further oxidize acetaldehyde that we were unable to identify at present (Table 3). The difficulty to elucidate this question rests on the fact that in these experiments we are measuring an intermediate metabolite, subject to a rapid degradation.

Acetaldehyde is a reactive molecule able to covalently bind to DNA, proteins and lipids, and other molecules such as GSH [38]. However, the production of reactive metabolites or processes by ethanol is not limited to the generation of acetaldehyde. It is known that in the case of other tissues such as liver, prostate, testes, ovaries, or mammary tissue, formation of free radicals occurs (e.g., 1-hydroxyethyl, hydroxyl or acetyl) [2–8]. That is of relevance because free radicals may lead to additional covalent binding processes and, further, to hydrogen abstraction reactions of oxidative nature in DNA, proteins, and lipids (e.g., lipid peroxidation) that might provoke oxidative stress if cellular antioxidant defenses are exceeded [9].

In the present studies we found that some manifestations of oxidative stress occur in the uterine horn, when animals were exposed to chronic alcohol drinking. In effect, we detected the formation of hydroxyl free radicals in the microsomal fraction of uterine horn tissue when incubations were made in the presence of NADPH generating system (Figure 2). The generation process is less intense than the equivalent one occurring in the liver microsomal fraction (Figure 2). We were not able to detect the production of 1-hydroxyethyl in the uterine horn tissue microsomal fraction in contrast to its neat detection in the case of the liver counterpart. We believe that this does not mean that its formation does not occur but that perhaps the intensity of the generation process is below the limit of our detection system.

An additional source of hydroxyl radicals was found for the case of the uterine horn cytosolic fraction in the presence of ethanol, when the incubation system included hypoxanthine as a cofactor (Figure 3). 

This uterine horn cytosolic hydroxyl radical generation process is significantly less intense than the one occurring, in the equivalent liver cytosolic fraction, in the presence of ethanol and hypoxanthine as cofactor (Figure 3). These hydroxyl radical formation processes were mediated by xanthine oxidoreductase as evidenced by their full inhibition by allopurinol, a specific inhibitor of this enzyme [37, 39]. The formation of the hydroxyl radicals might result from the xanthine oxidoreductase mediated reduction of O_2_ to H_2_O_2_ followed by a Haber Weiss transformation of the latter to the hydroxyl radicals [5, 37].

It is interesting to point out that the potential significance of these cytosolic free radical generating metabolic pathways might be favored under alcohol drinking circumstances, since it is very well known that the formation of purine degradations products is enhanced during alcohol drinking, and, consequently, the availability of the necessary cofactors for these cytosolic pathways of metabolism could be increased [40].

Decrease in cellular defenses might lead to the occurrence of oxidative stress at uterine horn level. That was suggested to occur in our experiments on the *t*-butylhydroperoxide-induced chemiluminescence test made in uterine horn homogenates from rats chronically drinking alcohol for 28 days. In effect, significantly higher levels of chemiluminescence were emitted by uterine horn homogenates challenged with *t*-butylhydroperoxide from alcohol treated rats than those from untreated control animals (Figure 4). This finding suggests that defenses against the oxidative challenge in alcohol treated animals could be significantly decreased [12, 41].

Part of the diminished defenses can be attributed to a low but significant decrease in GSH content and to a tendency but not significant increase in the GSSG content and decreases in the GSH/GSSG ratio. It was of relevance the observed decrease in the activity of GSPx. This might be of particular interest to explain the intense response of the uterine horn tissue to the *t*-butylhydroperoxide in the chemiluminiscence test reported in Figure 4. The significant role of GSH, GST and GSPx in the resistance of cells to oxidative damage, is well known [42].

The behavior of the uterine horn in response to alcohol drinking for 28 days was different from that of the liver. The liver evidenced an adaptative response leading to increased GSH levels and not of GRed activity. In addition, no significant changes in the level of GSSG, in the ratio of GSH/GSSG, or in the GST and GSPx activities were observed.

The generation of free radicals and a decrease in cellular defenses in the uterine horn tissue led to early indications of oxidative stress occurrence, such as those observed as decreases in the protein sulfhydryl content. However, no increases in protein carbonyl content were found after alcohol drinking for 28 days.

Several SH enzymes and even essential cofactors are known to have cellular relevance and might be the target of the early oxidative stress observed. Of particular significance would be the case of sulfhydryl enzymes containing critical SH groups linked to their function, typical examples being glyceraldehyde-3-phosphate dehydrogenase; pyruvate oxidase; hexokinase; creatine kinase; *δ*-aminolevulic acid dehydrogenase; DNA polymerase; and O-alkylguanine DNA alkyl transferase [43–48].

Acetaldehyde levels here reported to occur in the uterine horn tissue; the production of reactive free radicals and the promotion of oxidative stress might be, at least, partially involved in the generation of the significant alterations occurring in the uterine horn tissue ultrastructure.

However, other relevant causes of those significant changes might arise from hormonal changes provoked by alcohol drinking on, for example, ovarian tissue and in the increased levels of estrogen that it promotes [23, 34, 49]. These questions remain to be answered in future experiments.

## Figures and Tables

**Table 1:** Weight changes in uterine horn from rats receiving an alcohol containing liquid diet.

	Uterine horn (mg)	Body weight (grams)
Control	238.7 ± 39.0	216.5 ± 13.2
EtOH-treated	68.6 ± 18.0^a^	192.4 ± 31.6^b^

^a^ *P* < 0.05 when compared to EtOH-treated to Control.

^b^ *P* > 0.05 when compared to EtOH-treated to Control.

*n* = 10.

**Table 2:** Ethanol metabolism to acetaldehyde in the microsomal fraction of uterine horn tissue from rats receiving an alcohol containing liquid diet.

Experimental^a^	Acetaldehyde (nmol/mg protein)	
	Control	EtOH-treated^b^
−NADPH	1.67 ± 0.10	1.86 ± 0.10
+NADPH^c^	2.11 ± 0.07	2.67 ± 0.40

^a^Incubation mixtures containing microsomal fraction (0.17–0.19 mg protein/mL), 0.14 M ethanol, and, when indicated, NADPH generating system (0.45 mM NADP^+^, 4 mM d,l-isocitric acid trisodium salt, and 0.25 units of isocitric dehydrogenase) in KH_2_PO_4_ buffer were conducted for 1 hour at 37°C. Acetaldehyde was measured in the head space of each sample after adding 1 mL NaCl saturated solution. See Section 2 for details. Each result is the mean of three separate lots of pooled uterine tissue samples.

^b^ *P* < 0.05 when compared to Control versus EtOH-treated.

^c^ *P* < 0.05 when compared to −NADPH versus +NADPH.

**Table 3:** Ethanol metabolism to acetaldehyde in the cytosolic fraction of uterine horn tissue from rats receiving an alcohol containing liquid diet.

Experimental^a^	Acetaldehyde (nmol/mg protein)	
	Control	EtOH-treated
Ethanol only	1.39 ± 0.03	1.78 ± 0.05
Ethanol + NAD	1.67 ± 0.04^b^	0.43 ± 0.03^g^
Ethanol + NAD + HX	1.76 ± 0.13^c,e^	0.32 ± 0.01^g,h^
Ethanol + NAD + HX + allopurinol	0.13 ± 0.01^c,d,f^	0.27 ± 0.06^g,i,j^

^a^Incubation mixtures containing cytosolic fraction (1.4–1.6 mg protein/mL), 0.14 M ethanol, and, when indicated, 0.25 mM hypoxanthine (HX) and 0.3 mM NAD^+^ in STKM buffer were conducted for 1 h at 37°C. Acetaldehyde was measured in the head space of each sample after adding 1 mL NaCl saturated solution. See Section 2 for details. Each result is the mean of three separate lots of pooled uterine tissue samples.

^b^ *P* < 0.01 when compared to “Ethanol only” (Control).

^c^ *P* < 0.001 when compared to “Ethanol only” (Control).

^d^ *P* < 0.001 when compared to “Ethanol + NAD + HX” (Control).

^e^ *P* > 0.05 when compared to “Ethanol + NAD” (Control).

^f^ *P* < 0.001 when compared to “Ethanol + NAD” (Control).

^g^ *P* < 0.001 when compared to “Ethanol only” (EtOH-treated).

^h^ *P* < 0.05 when compared to “Ethanol + NAD” (EtOH-treated).

^i^ *P* > 0.05 when compared to “Ethanol + NAD + HX” (EtOH-treated).

^j^ *P* < 0.01 when compared to “Ethanol + NAD” (EtOH-treated).

**Table 4:** Protein sulfhydryl and protein carbonyl in uterine horn from rats receiving an alcohol containing liquid diet.

	nmol CO/mg protein^a^	nmol SH/mg protein^b^
Control	7.12 ± 1.16	11.32 ± 0.78
EtOH-treated	7.65 ± 1.08	9.71 ± 0.66

^a^Uterine horn tissue isolated from Control and EtOH-treated rats was homogenized in 0.15 M Tris-HCl/1 mM KH_2_PO_4_ (pH 7.4) and centrifuged at 600 ×g, and the supernatants were used to carbonyl determination as described in Section 2. Three samples per group were run, each consisting of a homogenate from a separate lot of pooled uterine horn tissue (five animals each). *P* > 0.05 (Control versus EtOH-treated).

^b^Uterine horn tissue isolated from Control and EtOH-treated rats was homogenized in 0.15 M Tris-HCl/1 mM KH_2_PO_4_ (pH 7.4) and centrifuged at 600 ×g and the supernatants were used to sulfhydryl determination as described in Section 2. Three samples per group were run, each consisting of a homogenate from a separate lot of pooled uterine horn tissue (five animals each). *P* < 0.05 (Control versus EtOH-treated).

**Table 5:** Glutathione levels in uterine horn and liver tissue from rats receiving an ethanol containing liquid diet.

Group	Total glutathione	Reduced glutathione *μ*mol/g wet tissue	Oxidized glutathione	GSH/GSSG mol/mol
Uterine horn				
Control	0.63 ± 0.02	0.57 ± 0.02	0.06 ± 0.03	9.00 ± 4.10
EtOH	0.59 ± 0.02^a^	0.50 ± 0.02^a^	0.09 ± 0.03^b^	5.40 ± 1.70^b^

Liver				
Control	3.94 ± 0.07	3.64 ± 0.06	0.30 ± 0.10	12.33 ± 4.02
EtOH	4.85 ± 0.05^a^	4.47 ± 0.02^a^	0.38 ± 0.05^b^	11.79 ± 1.52^b^

Glutathione levels were measured in liver and uterine horn tissues as described in Section 2. Each value is the mean ± S.D. from five separate tissue samples.

^a^ *P* < 0.05 when compared to control.

^b^ *P* > 0.05 when compared to control.

**Table 6:** Glutathione S-transferase activity (GST), glutathione reductase activity (GRed), and glutathione peroxidase activity (GPx) in uterine horn and liver tissue from rats receiving an ethanol containing liquid diet.

Group*	GST nmol conjugated GSH/min/mg protein	GRed nmol oxidized NADPH/min/mg protein	GPx nmol oxidized NADPH/min/mg protein
Uterine horn			
Control	19 ± 1	107 ± 10	201 ± 2
EtOH	31 ± 1^a^	106 ± 5^b^	177 ± 2^a^

Liver			
Control	148 ± 6	143 ± 6	677 ± 32
EtOH	152 ± 1^b^	223 ± 5^a^	664 ± 26^b^

*Glutathione S-transferase activity (GST) in uterine horn tissue and liver cytosol (3.6–3.9 mg protein/ml for uterine horn and 18.5–19.8 mg protein/mL for liver) was assayed as the thioether formation with CDNB and reading the absorbance at 412 nm. Glutathione reductase activity (GRed) in uterine horn tissue and liver cytosol (3.6–4.1 mg protein/mL for uterine horn and 18.2–19.5 mg protein/mL for liver) was determined by measuring the disappearance of NADPH at 340 nm. Glutathione peroxidase activity (GPx) in uterine horn tissue and liver cytosol (1.4–1.9 mg protein/mL for uterine horn and 8.9–11.2 mg protein/mL for liver) was analyzed in a reaction initiated by H_2_O_2_ and following the absorbance change at 340 nm. Each value is the mean ± S.D. from four separate samples (five animals each). See Section 2 for details.

^a^ *P* < 0.01 when compared to control.

^b^ *P* > 0.05 when compared to control.
